# Clofazimine Inhalation Suspension Demonstrates Promising Toxicokinetics in Canines for Treating Pulmonary Nontuberculous Mycobacteria Infection

**DOI:** 10.1128/aac.01144-22

**Published:** 2023-01-17

**Authors:** M. Kunkel, M. Doyle-Eisele, P. Kuehl, K. Rotermund, M. Hittinger, S. Ufer, M. Reed, M. Grant, T. Hofmann

**Affiliations:** a MarkAllen Consulting LLC, Lawrenceville, New Jersey, USA; b Lovelace Biomedical, Albuquerque, New Mexico, USA; c PharmBioTec Research and Development GmbH, Saarbrücken, Germany; d Swabian Tech LLC, Raleigh, North Carolina, USA; e COELUS LLC, Albuquerque, New Mexico, USA; f MannKind Corporation, Danbury, Connecticut, USA

**Keywords:** clofazimine, NTM infection, nontuberculous mycobateria, inhalation, nebulization, pre-clinical

## Abstract

Pulmonary nontuberculous mycobacteria (NTM) infection is recognized as a major global health concern due to its rising prevalence worldwide. As an opportunistic pathogen with increasing antibiotics resistance, prolonged systemic dosing with multiple antibiotics remains the primary treatment paradigm. These prolonged treatments, administered predominantly by oral or parenteral routes, often lead to systemic toxicity. A novel inhaled formulation of clofazimine may finally resolve issues of toxicity, thereby providing for improved NTM therapy. Clofazimine inhalation suspension was evaluated in canines to determine toxicity over 28 days of once-a-day dosing. The good laboratory practice (GLP) repeat dosing study evaluated low, mid, and high dosing (2.72 mg/kg and 2.95 mg/kg; 5.45 mg/kg and 5.91 mg/kg; and 10.87 mg/kg and 10.07 mg/kg, average male versus female dosing) of nebulized clofazimine over 30, 60, and 120 min using a jet nebulizer. Toxicokinetic analyses were performed on study days 29, 56, and 84. All three dose levels showed significant residual drug in lung tissue, demonstrating impressive lung loading and long lung residence. Drug concentrations in the lung remained well above the average NTM MIC at all time points, with measurable clofazimine levels at 28 and 56 days postdosing. In contrast, plasma levels of clofazimine were consistently measurable only through 14 days postdosing, with measurements below the limit of quantitation at 56 days postdosing. Clofazimine inhalation suspension may provide an effective therapy for the treatment of NTM infections through direct delivery of antibiotic to the lungs, overcoming the systemic toxicity seen in oral clofazimine treatment for NTM.

## INTRODUCTION

The antituberculous activity of clofazimine (CFZ) has been known since the 1950s, but its use as a riminophenazine antibiotic against Mycobacterium tuberculosis (MTB) and other related pulmonary diseases caused by nontuberculous mycobacteria (NTM) has been limited by systemic side effects due to its unique lipophilic characteristics. While its lipophilicity and anti-inflammatory properties made it ideal for treating leprosy, these same properties produce accumulation in skin, nerve, and fat tissues, leading to both mild and serious complications. Additionally, its poor oral uptake requires high doses that are associated with serious intestinal distress (1). The complications caused by clofazimine accumulation *in vivo* worked against its effectiveness toward MTB *in vitro*. Early animal studies, limited by dose toxicity, were inconclusive for systemic treatment of TB. Interest in CFZ diminished rapidly after the emergence of potent anti-TB agents like isoniazid and pyrazinamide in the 1960s. The drug is now largely reserved for combination treatment of drug-resistant TB, along with other antibiotics (such as linezolid). However, the emergence of multidrug-resistant, and now extensively drug-resistant (XDR), strains of TB and NTM have created renewed interest in this compound (1, 2), particularly due to research that suggests that riminophenazines inhibit mycobacterial energy metabolism, a potential target for slow-growing persistent NTM strains (3, 4).

Oral administration of CFZ frequently results in side effects that range from inconvenient to life-threatening. As a lipophilic molecule, skin/conjunctival discoloration is often the first effect seen, and while many side effects are self-correcting (such as ichthyosis, anorexia, diarrhea, corneal xerosis, and enlargement of lymph glands) after cessation of treatment, some, like gastrointestinal distress, are more serious, and in some cases fatal (5). At the very least, these side effects are inconvenient to the patient, and in many cases stigmatizing, due to the obvious skin discoloration.

CFZ is typically administered orally as a microcrystalline suspension with a lipid base to improve absorption. However, absorption can vary substantially when taken with food, with both the maximum observed concentration (*C*_max_) and terminal half-life being affected (6). CFZ in systemic circulation distributes into fatty tissues, mononuclear phagocytes (a beneficial characteristic), and all major organ systems (a decidedly nonbeneficial characteristic) (7). Combined with a long half-life, the accumulation of CFZ in multiple organ systems limits oral dosing of the drug and prevents the maintenance of drug concentrations in the lung at levels necessary to effectively inhibit NTM infection.

While clofazimine analogues (tetramethylpiperidyl derivatives) have been under investigation since the 1980s to reduce side effects, no riminophenazine analogue has been brought to market. In addition to research into analogues of clofazimine, several strategies have been employed to reduce gastrointestinal and other side effects by improving specific drug absorption and decreasing systemic drug distribution. The use of solid dispersions in nontoxic polymers, as well as liposomal and nanosuspensions, have demonstrated efficacy with decreased toxicity in mouse models, but none have progressed into clinical development ([Bibr B8][Bibr B9] 13).

While alternative manufacturing methods, such as spray drying, can improve aqueous solubility by creating particulates that can be administered either orally or by inhalation (14), we instead took advantage of the unique crystalline nature of CFZ. In a liquid formulation, the phagocytic uptake of CFZ crystals by monocytes appears to be a unique, but natural, means to control systemic distribution outside the lung (15).

We undertook a development process to reformulate CFZ into an inhaled dose formulation that could effectively deliver CFZ directly to the lung, while reducing the systemic toxicity seen in oral dosing. Our efforts to reformulate unmodified clofazimine have resulted in a novel aerosolizable liquid formulation (clofazimine inhalation suspension [CIS or MNKD-101]) that can be delivered to the lung using a standard mesh or jet nebulizer (16).

CIS delivers crystalline CFZ to the lung, in proximity to the resident macrophages. The macrophages can take up the drug through phagocytosis of the CFZ crystals, thereby limiting the systemic distribution of CFZ from the lung (15). Appropriate dosing, and drug uptake by the macrophages, would maintain CFZ in the lung at levels well above the MIC for all NTM. Our preclinical studies in mice and rats, and most recently in beagle canines, have shown conclusively that inhaled CIS delivers CFZ to the lung at concentrations well above the MIC for NTM infection, while maintaining a safety profile that is likely impossible with oral dosing. We also note that our previous unpublished 21-day single-dose PK study in rats verified that oral gavage CFZ dosing resulted in no CFZ accumulation in the lung, and no measurable lung CFZ concentrations just 6 h postdosing. This is critical, as recent evaluations of slowly and rapidly growing NTM isolates show that the MIC exceeded 4 μg/mL in 10% of rapidly growing species, and in 6% of slowly growing species (17). CIS therefore has the potential to significantly improve multidrug regimens for both nonresistant and resistant TB, and especially NTM infections, as a dosing regimen that achieves sustained lung CFZ concentrations >1.5 μg/g lung tissue, or >1,500 ng/g, would be adequate to ensure lung CFZ concentrations exceed the MIC for most M. avium complex and M. abscessus strains studied to date (18, 19).

While tuberculosis remains one of the most common mycobacterial infections that causes mortality, nontuberculous mycobacteria are increasingly responsible for complications of other lung disorders, like cystic fibrosis (CF). In fact, NTM infections are recognized as a major cause of pulmonary infection in CF (16, 20, 21). A hallmark of mycobacterial infection (both TB and NTM) is the formation of granulomas that limit the ability of antibiotics to reach the mycobacteria. This is the primary reason treatment for TB/NTM is long and rigorous. Current best practices in treatment call for administration of isoniazid, rifampicin, ethambutol, and pyrazinamide for 6 to 30 months (22). In almost all cases, treatments include combination therapy with multiple antibiotics (23).

We believe that CIS may provide an improved adjuvant therapeutic for NTM and other mycobacterial infections in combination with first-line, multidrug treatment regimens using azithromycin or clarithromycin, with rifampin, and ethambutol (24). With GLP dosing studies and primary toxicology studies completed, we present here the results from our latest GLP toxicology study in canines. The completion of this work is the final step before a first in human SAD/MAD safety study of this novel formulation for treating mycobacterial infections.

## RESULTS

### Pulmonary function and clinical observations.

There were no MNKD-101 [CIS]-related changes in tidal volume, respiratory frequency, or minute volume at any dose level that were statistically significant. Exposure to API produced similar results to the vehicle or air controls. Interdose differences were not considered test-article related because they were sporadic, transient, or within expected ranges of variation for beagle dogs undergoing similar study procedures.

There were no abnormal clinical observations reported for any animal at any evaluation point in the study. Body weight trends were analyzed for male and female dogs separately. No specific trend in weight gain or loss was seen due to sex. Weights during the 28-day dosing period showed no specific trend for any dose regimen, for males or females, and in general treated dogs retained a stable weight. Weight changes during the recovery period did not correlate to drug dosing, as both the air and vehicle control weights varied at least as much as the weights of dogs in any of the treatment groups, over the recovery period.

Ophthalmic examination of dogs before and after the 28-day treatment period were unremarkable and did not identify any issues related to treatment. Electrocardiogram results did not indicate any changes to heart rate, PR, QRS, or Qt interval. There were no obvious API-related abnormalities in rhythm or waveform morphology at any dose level compared to the vehicle group and to the predose period.

### Hematology and clinical chemistry.

Hematology, serum chemistry, and urinalysis parameters were obtained at necropsy, on study day [SD] 29 for animals designated for the main study, SD 56 for animals designated for 28-day recovery, and SD 84 for animals designated for 56-day recovery. Up through SD 29, there were 3 animals per gender/group for all measures, with 2 animals per gender/group for recovery measures at SD 56 and 84.

All notable hematology, clinical chemistry, and urinary measures were either unremarkable, incidental, or not considered related to the administration of MNKD-101. No parameter indicated toxicity of any kind, nor followed any dose relationship with MNKD-101.

### Clinical pathology.

At each designated necropsy (SD 29, 56, and 84), tissues were collected, weighed as applicable, and preserved for histopathologic examination. In general, visceral adipose tissue was examined throughout, and discoloration was found only in test article treated animals on SD 29. Gross observations related to the test article at the time of necropsy in SD 29 (main study) animals consisted of mild to moderate, diffuse, yellow discoloration of the adipose tissue in all high-dose animals, two mid-dose males and all females, and one low-dose female, but there were no correlating microscopic findings to explain the discoloration. No discoloration of the skin was noted for any animal.

Organ weights were collected and analyzed as absolute organ weight, organ to bodyweight ratio, and organ to brain weight ratio versus the air and vehicle controls. Some organ weight differences were statistically significant in males (lung, adrenal glands, heart, epididymides, and testes) and females (adrenal, liver, and spleen), but there were no correlating microscopic findings to explain the differences. Remaining organ weights were generally unremarkable relative to air and/or vehicle controls. There was often no consistency across sexes, or ratio measures, and the changes were typically of small magnitude. In addition, there were no test article related observations in any tissue examined.

Histopathological examination of tissues did not determine any finding of significance, with any reported lung or lymph node infiltrates being reported as largely mild or minimal in nature.

### Plasma toxicokinetics.

There were no quantifiable clofazimine plasma concentrations for males or females in the air or vehicle control groups (groups 1 and 2) at any time point in the study. Male and female animals in the MNKD-101 low-, mid-, and high-dose groups had quantifiable clofazimine concentrations at all time points sampled on SD 1 and 28 (see Table 1). On SD 42 (14 days of recovery from exposure), measurable concentrations were reported for all but one animal that was below quantitative limits (BQL). On SD 56, only one animal had a reportable CFZ concentration, while all the others were BQL. All samples were BQL on SD 84.

**TABLE 1:** Mean toxicokinetic parameters in dog plasma following a single dose on study day 1, or 28 consecutive daily doses on study day 28 for low-dose (group 3), mid-dose (group 4), or high-dose (group 5) animals

Study day	Group	Exposure	Dose (mg/kg)	*C*_max_ ± SE (ng/mL)	*t*_1/2 0–24_ (hr)	*t*_1/2 day 1–84_ (hr)
1	3	Low dose	0.68 male	16.2 ± 5.42	7.19	
			0.74 female	22.9 ± 1.79	8.99 ± 0.632	
1	4	Mid dose	1.36 male	43.1 ± 11.0	8.95 ± 1.51	
			1.48 female	33.2 ± 6.22	9.43	
1	5	High dose	2.72 male	112 ± 13.7	7.89	
			2.52 female	139 ± 30.1	7.23 ± 1.03	
28	3	Low dose	0.68 male	27.5 ± 5.06		NR
			0.74 female	67.1 ± 17.7		83.4
28	4	Mid dose	1.36 male	93.5 ± 19.9		106
			1.48 female	183 ± 42.9		78.2
28	5	High dose	2.72 male	271 ± 68.7		98.1
			2.52 female	241 ± 20.3		115

After exposure on day 1, the time of maximum concentration, or *T*_max_, was at the 0.5- or 6.0-h time point for the low-, mid-, and high-dose (groups 3, 4, and 5, respectively) males and females. After 28 consecutive daily doses on SD 28, *T*_max_ was at the 0.5-, 6.0-, or 12.0-h time point for female and male animals in all dose groups.

**FIG 1:**
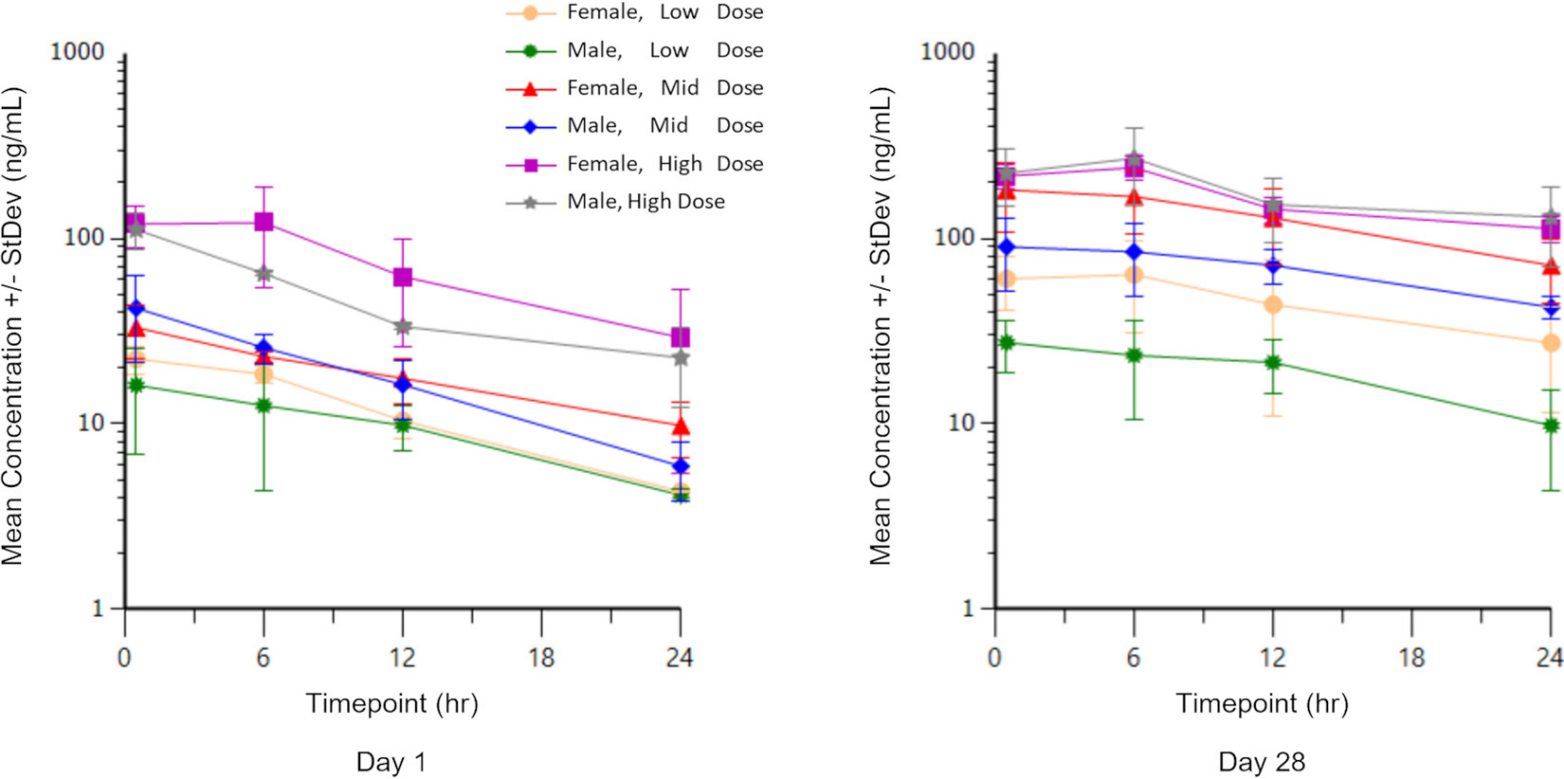
Semi-log plot of mean ± standard deviation clofazimine concentrations in dog plasma following a single dose (day 1) or 28 consecutive daily doses (day 28) of low-dose (group 3), mid-dose (group 4), or high-dose (group 5) MNKD-101. Initial evaluation was performed 0.5 h after dosing.

Mean peak clofazimine concentrations (*C*_max_) on SD 1 were 16.2 ng/mL, 43.1 ng/mL, and 112 ng/mL for males in the low-, mid-, and high-dose groups and 22.9 ng/mL, 33.2 ng/mL, and 139 ng/mL for females in the low-, mid-, and high-dose groups. After 28 consecutive daily doses of MNKD-101, on SD 28, mean *C*_max_ was 27.5 ng/mL, 93.5 ng/mL, and 271 ng/mL for males in the low-, mid-, and high-dose groups and 67.1 ng/mL, 183 ng/mL, and 241 ng/mL for females in the low-, mid-, and high-dose groups (Fig. 1).

Terminal elimination parameters could only be estimated for some animals on SD 1 and could not be estimated for any animals on SD 28. For animals with reportable terminal elimination parameters on SD 1, mean terminal elimination half-life of clofazimine was 7.19 h, 8.95 h, and 7.89 h for males in the low-, mid-, and high-dose groups and 8.99 h, 9.43 h, and 7.23 h for females in the low-, mid-, and high-dose groups.

Terminal elimination parameters also were calculated with the recovery time points of SD 42, 56, and 84. These terminal half-life values were not reported (NR), 106 h, and 98.1 h for males in the low-, mid-, and high-dose groups, respectively, and 83.4 h, 78.2 h, and 115 h for females in the low-, mid-, and high-dose groups, respectively. These are equivalent to a range of 3 to 5 days for males and females.

**FIG 2:**
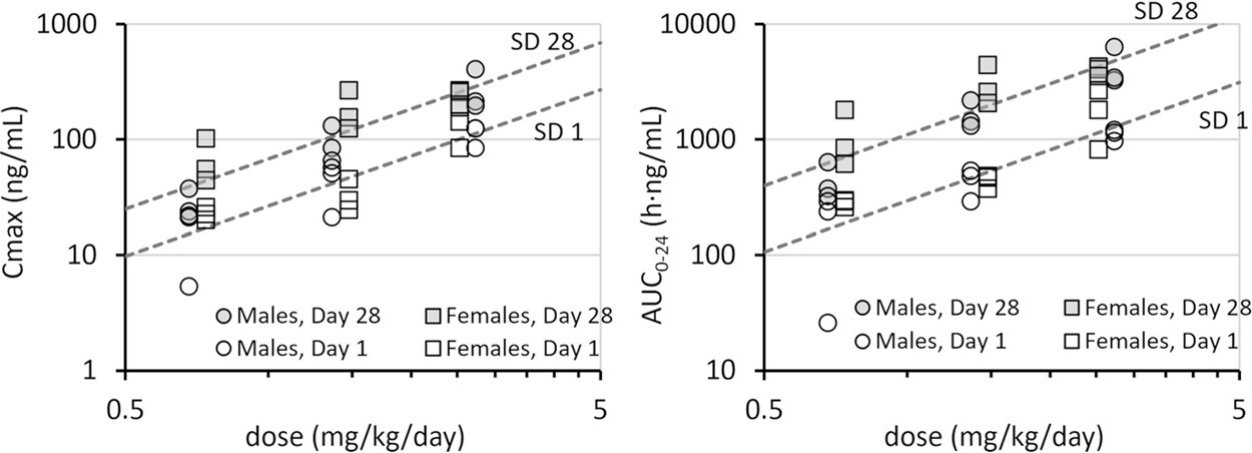
Log-log plot of dose dependence of plasma clofazimine PK in dog plasma. (Left panel) Plasma *C*_max_ was proportional to Dose^1.44^ on SD 1 and 28. *C*_max_ on SD 28 was approximately 2.56 times that on SD 1. (Right panel) AUC_0-24_ was proportional to Dose^1.47^ on SD 1 and 28. AUC_0-24_ on SD 28 was approximately 3.76 times that on SD 1.

In multiple linear regression on log-transformed variables, treatment on SD 28 had the effect of increasing the intercept in the model (*P* < 0.003) but did not affect the slope. The sex of the animal (and interactions) did not significantly affect the intercept or slope of the best-fit model. The mean ± standard error (SE) exponents for *C*_max_ (1.44 ± 0.14) and area under the concentration versus time curve (AUC_0–24_; 1.47 ± 0.16) indicate a supraproportional dose response because the values are not consistent with a hypothesized slope of 1 (*P* < 0.007). The geometric mean ratios of *C*_max_ and AUC_0–24_ on SD1 and SD28 were 2.56 (15% coefficient of variation [CV]) and 3.76 (17% CV), respectively (Fig. 2).

Mean accumulation ratios between SD 28 (after 28 consecutive daily doses) and SD 1 (after a single dose) for MNKD-101 dose-normalized (DN) AUC_0–24h_ were 5.39, 3.96, and 3.86 for males in the low-, mid-, and high-dose groups and 3.80, 6.92, and 2.90 for females in the low-, mid-, and high-dose groups. Accumulation ratios for DN *C*_max_ were 2.27, 2.54, and 2.44 for males in the low-, mid-, and high-dose groups and 3.08, 5.43, and 1.98 for females in the low-, mid-, and high-dose groups. All of the ratios indicate ≥2-fold accumulation in males and females after 28 consecutive daily doses.

**FIG 3:**
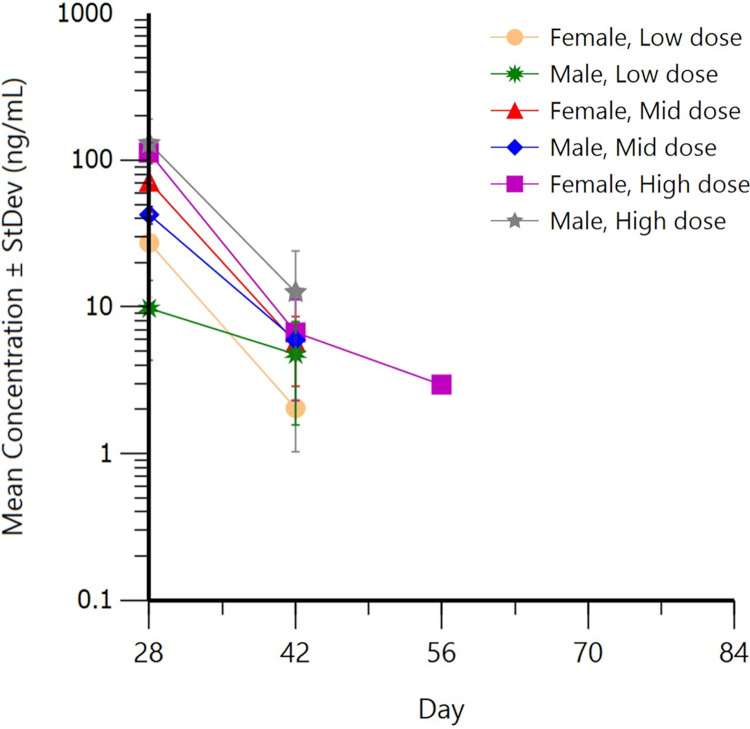
Semi-log plot of mean ± standard deviation clofazimine concentrations in dog plasma for recovery period after 28 consecutive daily doses (day 28) of low-dose (group 3), mid-dose (group 4), or high-dose (group 5) MNKD-101.

Plasma levels of MNKD-101 quickly decreased from maximum levels (SD 28) to BQL for nearly all animals by SD 56 (Fig. 3).

### Lung tissue toxicokinetics.

The toxicokinetics of MNKD-101 in lung tissue were evaluated from male and female beagle dogs following 28 consecutive daily doses (SD 29) and during the recovery period (SD 56 and SD 84). There were no quantifiable clofazimine concentrations in the air or vehicle control groups.

In general, lung concentration of clofazimine was dose dependent across males and females, with measured drug levels following dosing regimens at all time points except one (Table 2). Female lung drug levels were higher than male levels at SD 54 for the mid- and high-dose groups, but showed no specific trend at SD 29 or SD 84. Combined sex data clearly show residency of clofazimine at all time points in a dose-dependent manner.

**TABLE 2:** Group mean concentrations in male and female dog lung following 28 consecutive daily doses (SD 29) or recovery (SD 56 and 84) from low dose (group 3), mid dose (group 4), and high dose (group 5)

Sex	Group	Dose	Study day	*n*	Mean ± SD (ng/g)
Male	3	0.68	29	3	9450 ± 5940
			56	2	8600 ± 9190
			84	2	6320 ± 8030
	4	1.36	29	3	76700 ± 10800
			56	2	15300 ± 7790
			84	2	1680 ± 1910
	5	2.72	29	3	186000 ± 84500
			56	2	92400 ± 67400
			84	2	27300 ± 7000
Female	3	0.74	29	3	19800 ± 19500
			56	2	5670 ± 3400
			84	1	604 ± NA
	4	1.48	29	3	62300 ± 33900
			56	2	83500 ± 20800
			84	2	8710 ± 10200
	5	2.52	29	3	320000 ± 86700
			56	2	389000 ± 33200
			84	2	43100 ± 16900

**FIG 4:**
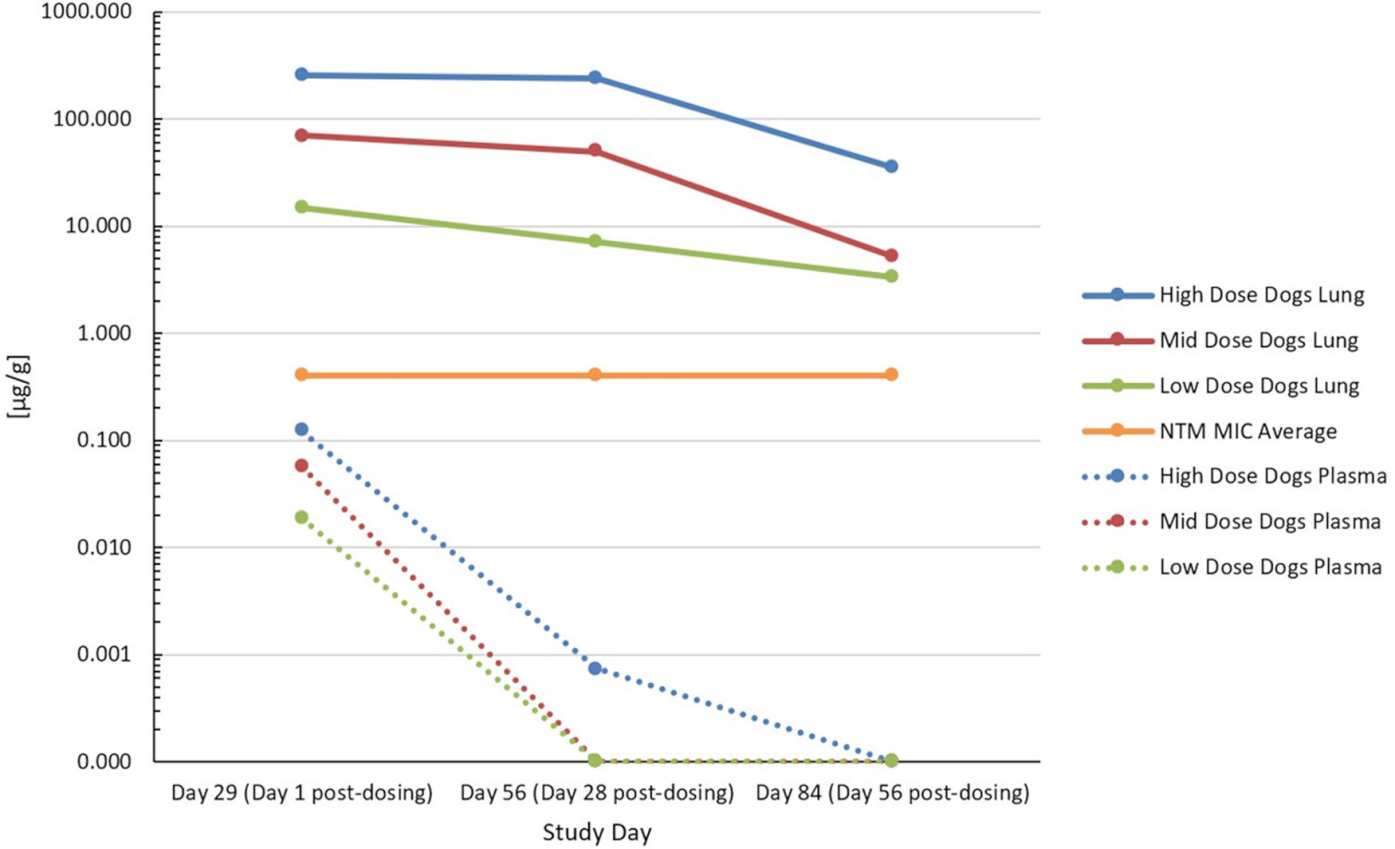
Lung and plasma levels of clofazimine postdosing. Zero values actually denote BQL.

Compared to plasma levels, lung concentrations of clofazimine more slowly decreased from SD 29 to SD 84, and remained well above the average MIC for NTM infections (Fig. 4). Terminal elimination half-lives were estimated only for low-dose females (10.9 days), mid-dose males (9.96 days), and high-dose males (19.8 days); other groups did not meet the reporting criteria.

Ratios for males compared to females for DN AUC_last_ were near unity for the low-dose groups, and DN AUC_last_ and DN *C*_max_ were near unity for mid-dose groups, indicating no notable (>2-fold) difference between sexes after 28 consecutive daily doses from SD 29 to SD 84. However, *C*_max_ for the low-dose group was nearly 2-fold increased, and both DN AUC_last_ and *C*_max_ were more than 2-fold increased for females compared to males in the high-dose group, so averages across males and females for toxicokinetic parameters are not presented since males and females were not comparable at all dose levels.

Dose proportionality as assessed by DN AUC_last_ and *C*_max_ ratios between dose groups showed much greater than dose-proportional increases across dose groups for males and females. Ratios between dose groups for AUC_last_ ranged from 1.57 to 3.44 for males and 2.86 to 8.27 for females. Ratios between dose groups for *C*_max_ ranged from 1.21 to 4.91 for males and from 2.11 to 5.76 for females.

## DISCUSSION

A primary premise for the CIS formulation is the improvement of residence time, and concentration, within the target organ, the lung, while reducing systemic accumulation, with the ultimate goal of reducing systemic toxicity. This GLP toxicokinetic study in beagle dogs provides confirmatory evidence that not only does CIS administration via inhalation reduce systemic CFZ accumulation, and nontarget organ toxicity, but also leads to superior deposition in the lung, at levels above the average MIC for NTM infections. Lung CFZ levels remained at concentrations well above the NTM MIC even 56 days post dosing, while systemic exposure to CFZ remained low, indicating that no reserve pools of drug were coming from tissue accumulation. This is an important finding that supports the observations that there were no obvious or measurable adverse effects from drug accumulation. For example, the fact that no animal was reported to have any skin discoloration is promising in terms of reducing a primary adverse effect of CFZ administration in humans, skin discoloration.

**FIG 5:**
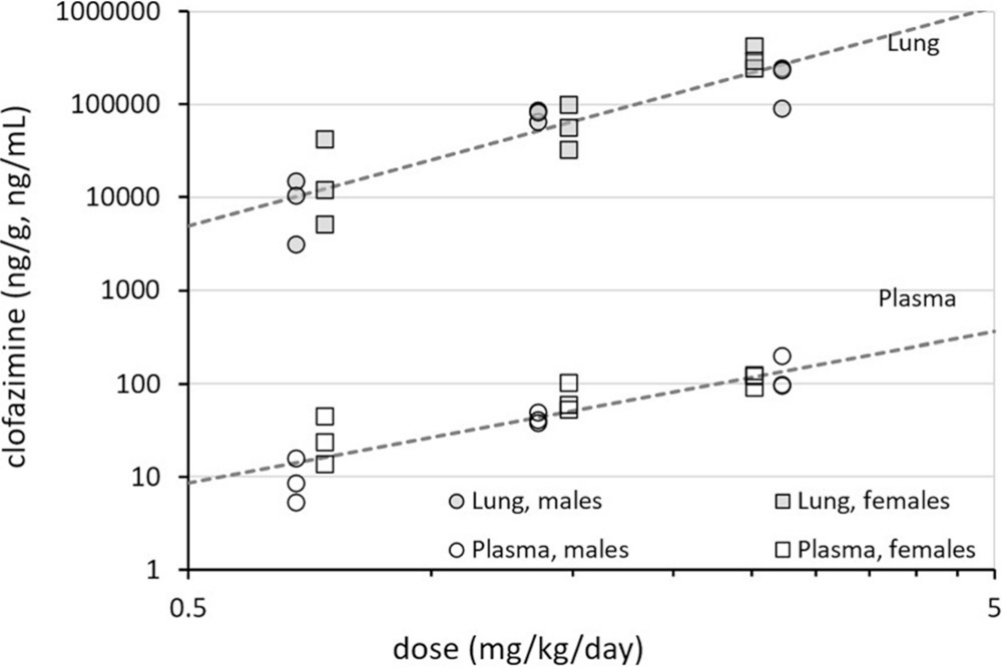
Clofazimine concentrations in lung and plasma on SD 29.

Importantly, clearance of CIS was also not dependent upon dose received, indicating that clearance mechanisms were not saturated by the dose levels used. This is clearly indicated by the fact that t_1/2_ showed no trends in any direction across dose groups, indicating that administering even higher doses of CFZ may be possible without untoward effects. Dose levels used in this study were determined from prior studies in mice and rats at doses up to 3.45 mg/kg/day, with a previous rat study also demonstrating very poor CFZ deposition in the lung from oral administration. Interestingly, the total lung fraction of CFZ in the low-dose group after 28 days of dosing appears to be primarily due to simple accumulation, while the highest dosing accumulation (exponential increase) indicates reduced transport of CFZ out of the lung, likely by incorporation into the lung resident macrophage population. This is particularly relevant for patients with reduced lung function from comorbidities that may result in irregular deposition of CIS. Combined with its very small inhaled particle size (1.5 to 2.5 mass median aerodynamic diameter), which should help reach deep within the lung, the increased residence time of CFZ through macrophage uptake may be a balancing factor against the possibility of irregular deposition from lung abnormalities. Concentrations of CFZ in plasma and lung tissue at necropsy illustrate the supraproportional behavior of the PK (Fig. 5).

Even at the highest level of CFZ delivered, no untoward physiology was seen in lung tissues directly, and few adverse events were seen systemically. At all doses, both liver and spleen showed no more than 0.1 μg CFZ per gram of tissue only 28 days postdosing, while adipose tissue showed a sharp linear decline to BQL by SD 56, demonstrating the rapid clearance of CFZ from nonlung tissues (Fig. S2). The ability to provide high-dose CFZ directly to lung tissues where it is needed, facilitates the use of CFZ in combination therapy with other antimycobacterial drugs, like ethambutol, macrolides, and rifampin. Considering the potential side effects of multidrug regimens, any reduction in complications from CIS use as an adjuvant therapeutic with standard-of-care NTM treatment would be beneficial to ameliorate the more serious psychological effects in patients using CFZ, due to potential depression, driven by skin discoloration, gastrointestinal derangement, and cardiac arrhythmias (contraindications for Lamprene use [25]).

With nebulizers being a simple, cost-effective, and reusable means to deliver CFZ to the lung, in a home or hospital setting, CIS dosing for NTM infections may encourage patient use, and adherence, in long-term adjunctive therapy. Our study raises the possibility of attaining very high, sustainable clofazimine levels in the lung, without increasing side effects, and directly supports dosing calculations and regimens for a first-in-human SAD/MAD study of CIS.

## MATERIALS AND METHODS

### Clofazimine inhalation suspension (CIS) for nebulization.

The MNKD-101 (CIS) formulation consists of clofazimine particles (20 mg/mL) suspended in 0.9% saline, with polysorbate 80 (0.5% vol/vol) to stabilize the suspension. The drug product is stable at room temperature for 1 year. Vehicle consisted of sterile 0.5% Polysorbate 80 (Hx2) in 0.9% saline. The formulation was optimized and tested *in vitro* at PharmBioTec to reduce animal experiments (data not shown).

### Study design.

All GLP canine study activities were performed by Lovelace Biomedical Research Institute (LBRI) under an IACUC-approved protocol. Five groups of Beagle dogs were divided into study groups (Table 3) and exposed once daily by face-mask inhalation to filtered air (group 1), vehicle (group 2; 0.5% polysorbate in saline), or MNKD-101 (groups 3 to 5) for 28 consecutive days. The low, mid, and high doses (groups 3, 4, and 5) were exposed to an average aerosol concentration of 0.209 mg/L for 30 min, 60 min, and 120 min, respectively. The 28-day exposure period was followed by 28- and 56-day no-exposure recovery periods (recovery study; SD 56 and 84, respectively). Blood was collected for toxicokinetic (TK) analysis from animals after both the first day of dose administration (day 1; 0.5 h [± 5 min], 6 h [± 30 min], 12 h [± 30 min], 24 h [± 1 h]) and the last day of dose administration (SD 28), and at SD 41, 56, and 84, as indicated in Table 3. Main study animals were necropsied 1 day post final exposure (SD 29), while two male and two female recovery study animals were each necropsied on SD 56 and SD 84.

**TABLE 3:** Study groups, exposure, and collection points

Group	Exposure	Target aerosol exposure conc. (mg/L)	Exposure duration (minutes)	Deposited lung dose, 25% deposition fraction (DF) (mg/kg)	TK blood collection study Days, time points, and sample sizes			
					Main study		Recovery study	
					Day 1, 28		Day 42, 56, 84	
1	Air control	Not applicable	120	0	3 male	3 female	4 male	4 female
2	Vehicle control	0.18 mg/L total aerosol conc.	120	0	3 male	3 female	4 male	4 female
3	Low-dose MNKD-101	0.40 mg/L total aerosol conc. (estimated 0.209 mg/L clofazimine aerosol conc.)	30	0.68 M / 0.74 F (0.71)	3 male	3 female	4 male	4 female
4	Mid-dose MNKD-101		60	1.36 M / 1.48 F (1.42)	3 male	3 female	4 male	4 female
5	High-dose MNKD-101		120	2.72 M / 2.52 F (2.62)	3 male	3 female	4 male	4 female

### Aerosol dosing.

Aerosolized CIS was delivered to each dog by way of a 6-port aerosol exposure system using three Micro Mist compressed air jet nebulizers (Fig. S1). All animals were conditioned to the face masks and restraint system before study. The exposure system and aerosol characteristics of the API and vehicle, including target aerosol concentration, concentration homogeneity, concentration repeatability, and aerosol size distribution, were confirmed by LBRI prior to study. Dosing for each dog was measured for every dose delivered throughout study, and calculated against individual dog weights (Equation 1 in supplemental file) (26, 27).

### Tissue and plasma analysis.

Blood samples were immediately processed or held on wet ice for no more than 2 h before being processed to plasma by centrifugation (1,300 *g*, 2 to 8°C, ≥10 min), with plasma separated into appropriately labeled vials and stored frozen (−70 to –90°C) until CFZ analysis.

Main study animals (3M/3F per time point) had blood samples collected at the following time points postexposure on SD 1 and 28: 0.5 h (± 5 min), 6.0 h (± 30 min), 12 h (± 30 min), and 24 h (± 1 h). Recovery animals (2M/2F per recovery time point) from each group had blood collected on SD 42, and prior to euthanasia on SD 56 or 84. The sample size for each group, sex, and time point is described in Table 3. Actual collection times were documented, and all animals in this study underwent scheduled sampling at their respective time points.

### LC-MS determination of CIS.

Clofazimine was extracted by protein precipitation from dog plasma. Clofazimine from tissues was first extracted by homogenization of tissues with a Bead Rupter during the extraction process. Reversed-phase high-pressure liquid chromatography (HPLC) separation was achieved with a Waters Acquity UPLC BEH C18 (2.1 × 50 mm, 1.7 μm) column on a Shimadzu Nexera X2 UHPLC system. Subsequently, MS/MS detection (Sciex Triple Quad 5500) was set at mass transitions of *m/z* 473.2→431.1 for clofazimine, and 480.2→432.1 for clofazimine-d7, respectively, in positive mode. Retention time and peak area were determined by Analyst Data Acquisition/Processing software (version 1.6.3). Analyte concentrations were obtained from a calibration curve constructed by plotting the peak area versus the nominal concentration using Analyst.

### Pathology.

Tissues were collected, examined, and weighed as applicable, and representative samples were preserved for histopathology. Eyes with optic nerves, testes, and epididymides were fixed in Modified Davidson’s fluid; other tissues were fixed in 10% neutral buffered formalin (NBF). Lung lobes were instilled via major airway(s) with NBF (to approximate physiologic full lung volume at 25 cm hydrostatic pressure); the major airway(s) used for instillation were closed, and the lung/lobe immersed in NBF for fixation.

Tissues were paraffin embedded, sectioned, and stained with hematoxylin and eosin for microscopic examination. Histopathologic examination was conducted in a “read down” fashion: i.e., all tissues and gross lesions were examined for animals exposed to filtered air control, vehicle control, or MNKD-101 via face-mask inhalation at the high dose. Only respiratory tissues (lungs, tracheobronchial lymph node, pharynx, larynx, trachea, and nose/turbinates) and gross lesions were examined in low- and mid-dose animals.

Visceral fatty tissue was evaluated for discoloration during necropsy for each animal. Any skin discoloration was noted by external examination by the attending pathologist during necropsy. Several skin samples were taken from the inguinal region for evaluation. All findings for a given tissue were graded subjectively and semiquantitatively by a single pathologist on a scale of 1 to 5 (1 = Minimal, 2 = Mild, 3 = Moderate, 4 = Marked, 5 = Severe).

### Analysis.

Clinical observations were descriptive, while numerical evaluations (means and standard deviations) were used for all other parameters where possible. Toxicokinetic parameters were estimated for blood plasma using Phoenix WinNonlin version 8.3 software (Certara, L.P.) with a noncompartmental analysis (NCA) consistent with aerosol administration (extravascular model) on each subject at each time point. NCA was only performed if there were quantifiable concentrations at two consecutive time points. Concentration values below the lower limit of quantitation of 2.00 ng/mL for plasma were labeled as below quantitative limits (BQL). These BQL values were treated as missing and excluded from calculation of descriptive statistics and toxicokinetic analysis.

Concentrations were used with full precision to three significant figures as received from bioanalytical data. Individual concentrations for clofazimine in plasma were collected by subject and time point for males and females separately. The area under the concentration versus time curve (AUC_0–24h_) for each subject on SD 1 and 28, from time zero to the time point at which the last quantifiable concentration was observed, was calculated with the linear-up log-down interpolation method. Parameters were also estimated for time of maximum observed concentration (*T*_max_), dose-normalized AUC_last_, the maximum observed concentration (*C*_max_), and dose-normalized *C*_max_. The terminal elimination phase of each concentration versus time curve was identified using at least the final three observed concentration values. The slope of the terminal elimination phase was determined using log regression with uniform weighting. Parameters derived from the terminal elimination phase were reported if they passed the reporting criteria: the coefficient of determination (R^2^) was greater or equal to 0.8, and the extrapolation of the AUC to infinity was less than or equal to 20% of the total area. The average doses by group and sex, assuming 25% deposition fraction, are transcribed from the aerosol report.

In addition, data were averaged for each gender and group to include the time points sampled from the recovery period on SD 42, 56, and 84. Since blood was collected from different animals for the main study (SD 1 and 28) and recovery time points, individual NCA parameters could not be calculated. Instead, gender/group average concentrations were used to include both sets of data.

### Statistics.

Arithmetic mean, standard deviation (SD), and sample size of clofazimine concentrations in plasma were calculated for each group and time point in Phoenix for males and females separately. Coefficient of variance (CV%) was calculated for NCA parameters, and geometric mean and geometric SD were calculated for accumulation ratios in Phoenix. Sex ratios were calculated in Phoenix by dividing dose-normalized AUC_last_ and dose-normalized *C*_max_ for male animals by the same parameters for female animals. Accumulation ratios were calculated in Phoenix by dividing dose-normalized AUC_last_ and dose-normalized *C*_max_ after 28 consecutive daily doses (day 28) by the same parameters after a single dose (day 1) for male and female animals. Dose-proportionality ratios were calculated in Phoenix by comparing dose groups pairwise for dose-normalized AUC_last_ and dose-normalized *C*_max_ values after 28 consecutive daily doses (SD 28) or after a single dose (SD 1) for male and female animals.

The dose dependence of *C*_max_ and AUC_0-24_ was evaluated as a power law (i.e., *C*_max_, AUC_0–24_ ~ Dose^n^) was evaluated for pooled data. The effects of sex, treatment day, and interactions between sex and treatment day were estimated by multiple linear regression on log-transformed coordinates. A full model was run to identify significant terms (*P* < 0.05), and the reduced model was then run to obtain estimates of the parameters. The regression was done in Excel.
