# Supplementary material for: Clofazimine Inhalation Suspension Demonstrates Promising Toxicokinetics in Canines for Treating Pulmonary Nontuberculous Mycobacteria Infection
Source: Antimicrob Agents Chemother. 2023 Jan 17;67(2):e01144-22. doi: 10.1128/aac.01144-22 (PMC9933692; doi:10.1128/aac.01144-22)

Supplemental Data:

Figure S1 – Diagram of Lovelace Canine Aerosol Exposure System

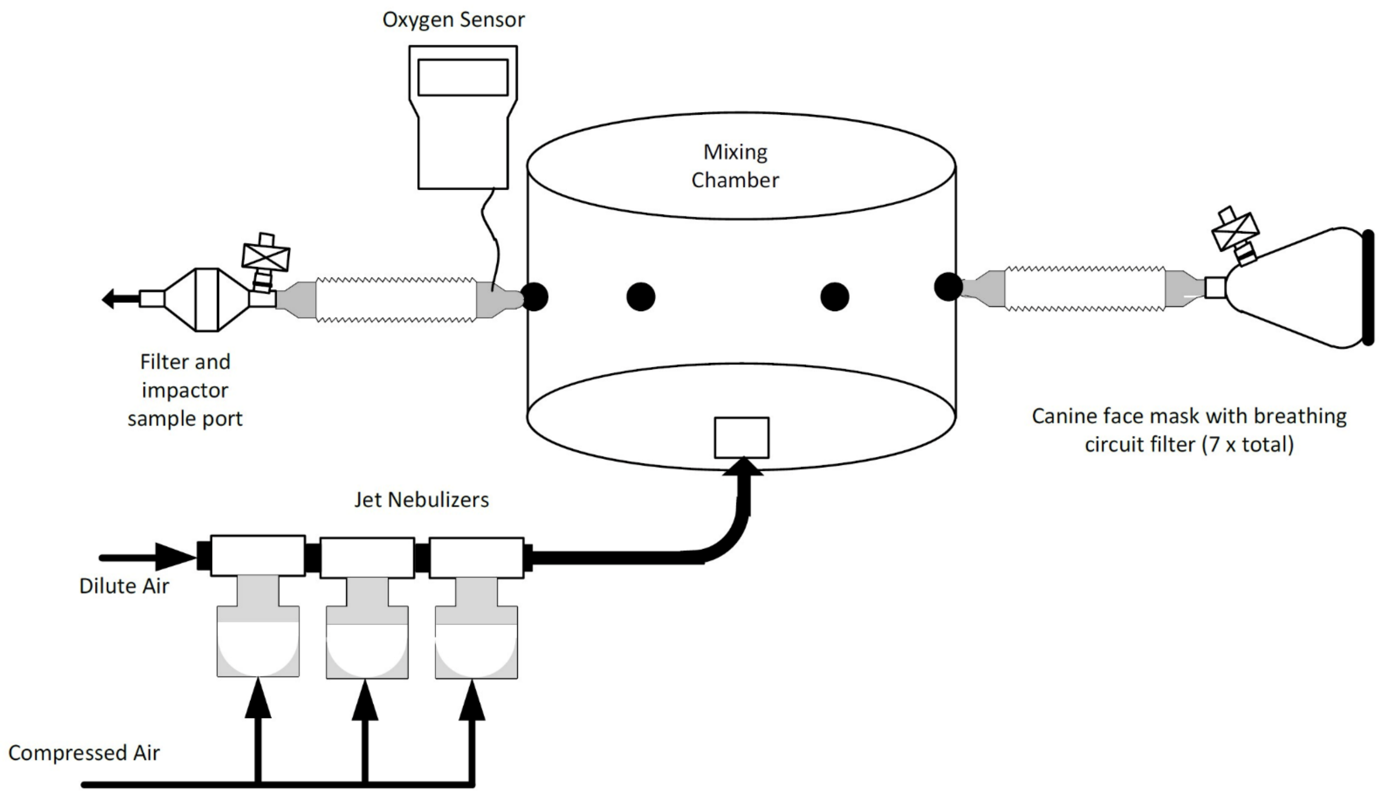

<u>Equation 1 – Aerosol dose calculation</u>

API aerosol doses were calculated using Equation 1 below based on the target API aerosol concentration of 209 µg/L from the aerosol method development using the 8-port chamber exposure system, to achieve the targeted pulmonary deposited doses, assuming an average canine weight of 8 kg.

$$\text{Eq1.} \qquad DD(\mu g/kg) = \frac{AC(\mu g/L) \times RMV\ (L/min) \times DF \times T(min)}{BW(kg)}$$

Where:

- Deposited Dose = (DD) µg/kg; Exposure duration = T (min.)
- Respiratory minute volume (RMV) = 0.608 x BW0.852 [26]
- Average Aerosol exposure concentration (AC) = aerosol concentration (µg/L)
- Deposition Fraction (DF) = assumed deposition fraction of 25% for deposited dose and
- assumed deposition fraction of 100% for delivered dose [27]
- BW = average body weight of individual animals during exposure phase (kg)

Figure S2: Liver, Spleen and Adipose levels of CFZ in comparison to lung residence time.

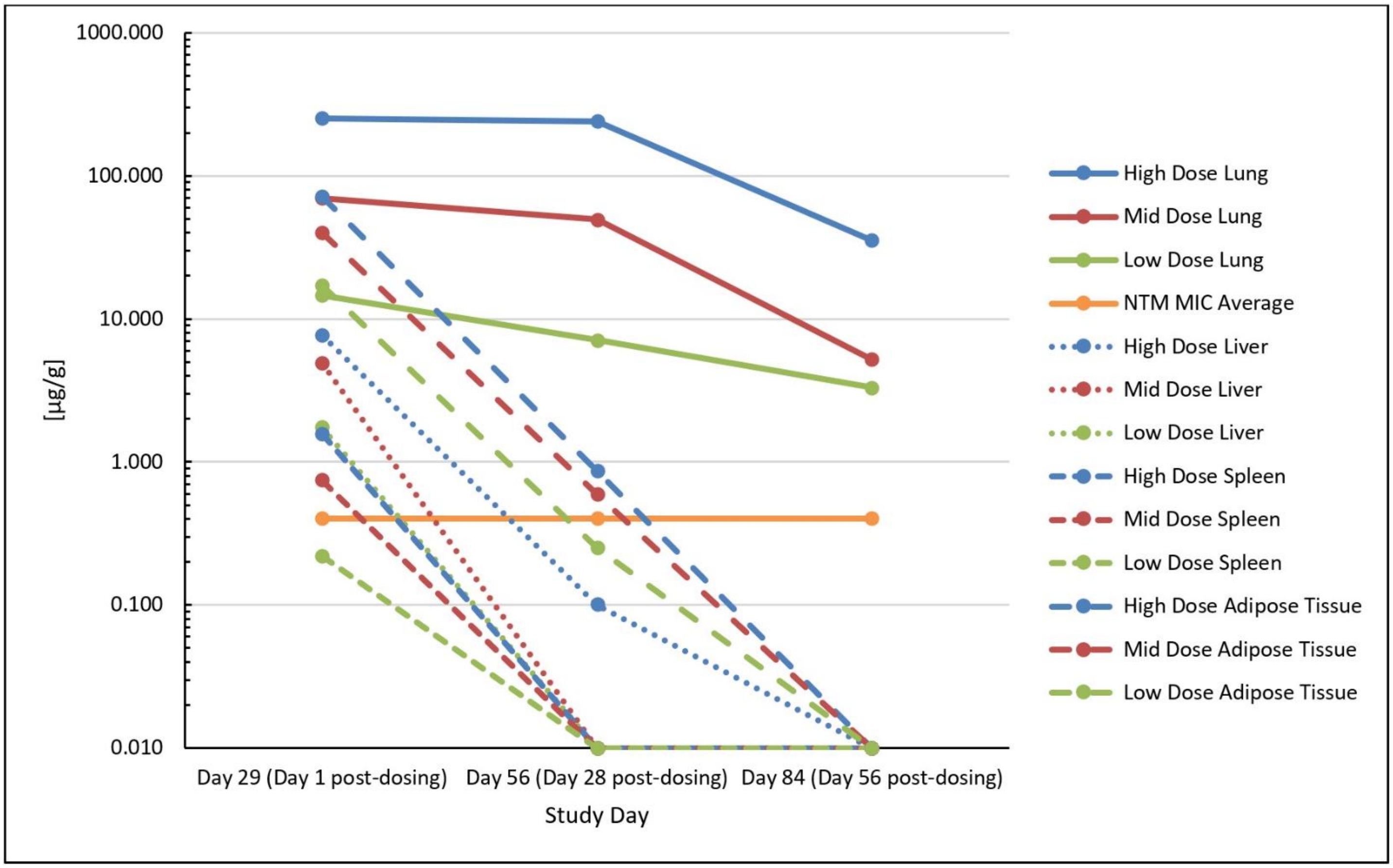

Supplement: Supplemental file 1 — Supplemental material. Download aac.01144-22-s0001.pdf, PDF file, 0.5 MB [file aac.01144-22-s0001.pdf]
